# Imaging glioma biology: spatial comparison of amino acid PET, amide proton transfer, and perfusion-weighted MRI in newly diagnosed gliomas

**DOI:** 10.1007/s00259-019-04677-x

**Published:** 2020-01-17

**Authors:** S. Schön, J. Cabello, F. Liesche-Starnecker, M. Molina-Romero, P. Eichinger, M. Metz, I. Karimov, C. Preibisch, J. Keupp, A. Hock, B. Meyer, W. Weber, C. Zimmer, T. Pyka, I. Yakushev, J. Gempt, B. Wiestler

**Affiliations:** 1 Department of Neuroradiology, Klinikum Rechts der Isar, Technical University of Munich, Ismaninger Str. 22, 81675 Munich, Germany; 2 Department of Nuclear Medicine, Klinikum Rechts der Isar, Technical University of Munich, Munich, Germany; 3 Department of Neuropathology, Institute of Pathology, Technical University of Munich, Munich, Germany; 4 Image-based Biomedical Modeling, Technical University of Munich, Munich, Germany; 5 Philips Research, Hamburg, Germany; 6 Philips Health Systems, Zurich, Switzerland; 7 Department of Neurosurgery, Klinikum Rechts der Isar, Technical University of Munich, Munich, Germany

**Keywords:** Amide proton transfer-weighted (APTw) imaging, O-(2-[18F]fluoroethyl)-l-tyrosine (FET) PET, Cerebral blood volume (CBV), Glioblastoma

## Abstract

**Purpose:**

Imaging glioma biology holds great promise to unravel the complex nature of these tumors. Besides well-established imaging techniques such O-(2-[18F]fluoroethyl)-l-tyrosine (FET)-PET and dynamic susceptibility contrast (DSC) perfusion imaging, amide proton transfer–weighted (APTw) imaging has emerged as a promising novel MR technique. In this study, we aimed to better understand the relation between these imaging biomarkers and how well they capture cellularity and vascularity in newly diagnosed gliomas.

**Methods:**

Preoperative MRI and FET-PET data of 46 patients (31 glioblastoma and 15 lower-grade glioma) were segmented into contrast-enhancing and FLAIR-hyperintense areas. Using established cutoffs, we calculated hot-spot volumes (HSV) and their spatial overlap. We further investigated APTw and CBV values in FET-HSV. In a subset of 10 glioblastoma patients, we compared cellularity and vascularization in 34 stereotactically targeted biopsies with imaging.

**Results:**

In glioblastomas, the largest HSV was found for APTw, followed by PET and CBV (*p* < 0.05). In lower-grade gliomas, APTw–HSV was clearly lower than in glioblastomas. The spatial overlap of HSV was highest between APTw and FET in both tumor entities and regions. APTw correlated significantly with cellularity, similar to FET, while the association with vascularity was more pronounced in CBV and FET.

**Conclusions:**

We found a relevant spatial overlap in glioblastomas between hotspots of APTw and FET both in contrast-enhancing and FLAIR-hyperintense tumor. As suggested by earlier studies, APTw was lower in lower-grade gliomas compared with glioblastomas. APTw meaningfully contributes to biological imaging of gliomas.

## Introduction

Traditionally, tumor imaging has focused on visualizing anatomy. Paralleling increasing insights into the complex biology of gliomas, the question of how biology is reflected in the imaging phenotype has gained a significant amount of interest in recent years. Among the most extensively studied imaging modalities for this purpose are O-(2-[18F]fluoroethyl)-l-tyrosine (FET)-PET and dynamic susceptibility contrast (DSC) perfusion imaging:

Using FET as a tracer, PET can visualize the amino acid uptake in gliomas and thus metabolically active tumor cells [1]. Several studies have demonstrated the clinical utility of FET-PET for preoperative grading [2] and biopsy planning [3] as well as for differentiating tumor progression from therapy-associated changes [4]. MR-based DSC perfusion provides evidence of neoangiogenesis [5]—a hallmark of malignant gliomas—and thereby also helps to distinguish between gliomas of different WHO grade and malignancy [6]. In addition, DSC has been shown to reflect differences in angiogenic pathways between isocitrate dehydrogenase (IDH) mutant and wild type gliomas [7] and to help predict patient survival [8] and response to anti-angiogenic therapy (bevacizumab) [9].

Amide proton transfer–weighted (APTw) imaging is a relatively novel MRI technique that relies on the constant dissociation and transfer of amide-bound hydrogen atoms into the surrounding water. By first saturating the amide-bound hydrogen, and then measuring the decrease in free water signal (due to the transfer of saturated hydrogen atoms into the surrounding free water pool), APTw imaging semi-quantitatively reflects the concentration of endogenous proteins and peptides. Two studies have found a good correlation between APT-weighted signal values and protein profiles [10] and tissue proliferative index (Ki-67) [11], respectively. APTw imaging has been shown to readily differentiate between gliomas of different WHO grades [12] and has shown promise to aid in the differentiation of tumor progression from therapy-associated changes [13, 14].

Integrating information from different techniques and modalities has helped to further decipher the complex interplay of biological processes in gliomas [15]. A few studies have already investigated the added value of APTw to diffusion- and perfusion-weighted imaging [16] or methionine PET [17].

In gliomas, a recent study surprisingly reported no correlation between APTw and FET [18]. Given the partially overlapping biological processes captured by these imaging modalities, these findings motivated us to investigate the synergism of APTw, FET, and CBV and how well they capture cellularity and vascularisation in a series of patients with newly diagnosed gliomas, both in *IDH* mutant and wild type tumors.

## Materials and methods

### Patients

All patients were part of a prospective observational glioma cohort from October 2017 to June 2018, which was approved by our local Institutional Review Board, and gave written informed consent. All patients with a newly-diagnosed glioma and complete preoperative imaging (PET and MRI) were included in this study. Neuropathological diagnosis was made according to the 2016 WHO classification [19].

### Image acquisition

Static PET data were acquired for 15 min starting 25 min post-injection, in a Siemens (Erlangen, Germany) Biograph mCT (vVG51C) in 24 patients, and in a Siemens Biograph mMR (vE11) in 24 patients.

MR imaging was performed on a Philips (Best, The Netherlands) 3 T scanner (Achieva or Ingenia). Our MR protocol included an isotropic T1 (voxel size 1mm^3^, TE = 4 ms, TR = 9 ms) before and after contrast, isotropic FLAIR (voxel size 1mm^3^, TE = 269 ms, TR = 4800 ms, TI = 1650 ms), axial T2 (voxel size 0.36 × 0.36x4mm^3^, TE = 87 ms, TR = 3396 ms), 3D APTw (fast spin echo, voxel size 0.9 × 0.9 × 1.8mm^3^, TE = 7.8 ms, TR = 6 s, RF saturation pulse train B_1,rms_ = 2μT, T_sat_ = 2 s, duty-cycle 100%, 9 volumes ω = ±3.5 ppm ± 0.8 ppm and reference ω_0_ = −1560 ppm, intrinsic B_0_ correction [20], MTR asymmetry at +3.5 ppm as APT-weighted = APTw contrast), pre- and post-contrast T1-TFE (voxel size 1mm^3^, TE = 4 ms, TR = 9 ms) as well as DSC perfusion (voxel size 1.75 × 1.75x4mm^3^, TE = 40 ms, TR = 1547 ms, Flip Angle = 75°, 80 dynamics).

### Image processing

PET data were corrected for decay, scatter and attenuation, using a low dose CT as attenuation map in the Biograph mCT and a two-point Dixon-based attenuation map, including bone from an atlas, in the case of the Biograph mMR [21]. Next, PET data were reconstructed using the OP-OSEM algorithm (3 iterations and 24 subsets) with the Siemens off-line reconstruction e7 tool, in a matrix size of 344x344x109 voxels (2.4 × 2.4 × 2.0 mm^3^ voxel size) for the mCT and 344x344x127 voxels (2.1 × 2.1 × 2.0 mm^3^ voxel size) for the mMR, and a post-reconstruction Gaussian filter of 3.0 mm kernel size. Neither time-of-flight (TOF) nor point-spread-function (PSF) were considered in the reconstructions to avoid introducing additional differences between the images obtained from both scanners. Standardized uptake values (SUV) were calculated from patient weight and injected activity. For background correction, a circular ROI was placed in gray and white matter of the non-tumor-bearing hemisphere by IV and BW [22].

Processing of DSC data and calculation of cerebral blood volume (CBV) maps used custom programs in MATLAB R2016a (MathWorks, Natick, MA, USA). Spatial co-registration of different modalities and segmentation of anatomical images for gray matter (GM), white matter (WM) and CSF were conducted using SPM12 (www.fil.ion.ucl.ac.uk/spm) with standard parameter settings. Leakage-corrected CBV values were obtained using a reference curve approach and numerical integration [23–25]. Relative CBV (rCBV) values were calculated by assuming healthy WM values of 2.5% [26].

Post-processing of APTw images followed the vendor’s standard implementation.

### Stereotactic biopsies and neuropathological analysis

For 10 glioblastoma patients, spherical volumes of interest (VOI) of 1 cm diameter were defined on the preoperative images in consensus with the operating neurosurgeon. Using a cranial navigation system (Varioguide, Brainlab AG, Munich, Germany; stereotactic accuracy approximately 3 mm), several biopsies per patient were obtained and sent in 10% buffered formalin to the Department of Neuropathology for histological evaluation.

After formalin fixation and paraffin embedding, hematoxylin and eosin staining was performed on 2 m-thick slices. Analysis of cellularity and neovascularization was conducted by a neuropathologist not familiar with the results of imaging analysis. For cellularity, cells were counted in 1/4 high power field (ocular × 10, objective × 40) of three regions of each biopsy. Regions were randomly chosen, necrotic areas were excluded. Neovascularization was scored from 0 to 2, 0 meaning no, 1 few, and 2 many vascular proliferates compared to gross tumor area.

### Image and statistical analysis

All images and parameter maps from a single patient were spatially aligned to the non-enhanced T1 image using a rigid, mutual information-driven registration with the open-source ANTs software (https://stnava.github.io/ANTs/) and re-sampled to 2 mm isotropic resolution. Tumors were semi-automatically segmented into two mutually exclusive volumes, contrast-enhancing tumor (CET) and FLAIR-hyperintense tumor (FHT), using a threshold-based segmentation in ITK-Snap [27]. All resulting segmentations were manually corrected where necessary by SS (8 years of experience), with a special emphasis on excluding cystic / necrotic areas from the final segmentations.

To quantify the spatial overlap of hot-spot areas delineated by the different modalities, we threshold APTw, FET and CBV parameter maps by using the following cut-offs: APTw > 1.79 [14], Tumor-Background-Ratio > 1.6 for FET [28], and rCBV > 5.6 [29]. Next, hot-spot volumes and Dice scores were calculated separately in CET and FHT through simpleITK filters (http://www.simpleitk.org/). In brief, the Dice score (ranging from 0 to 1) quantifies how well two segmentations overlap. Given two binary segmentation masks X and Y, the Dice score D is calculated as $D=\frac{2\ast (XuY)}{X+Y}$. In both CET and FHT, we additionally calculated median APTw and CBV values in FET-positive and FET-negative areas.

Median scores were compared using Wilcoxon’s rank-sum test, and *p* value < 0.05 was considered statistically significant. Calculations were carried out using Python version 3.6 and R version 3.5. All scripts are available upon request from the corresponding author.

## Results

### Patient cohort

In total, our cohort comprised 46 patients **(**Table 1): 31 patients were diagnosed with an *IDH* wild type WHO grade IV glioblastoma, 12 patients with an *IDH* mutant, 1p/19q codeleted oligodendroglioma (WHO grade II/III) and 3 patients with an *IDH* mutant, 1p/19q intact astrocytoma (WHO grade III). Given that *IDH* status separates gliomas into two biologically distinct diseases, we grouped *IDH* mutant tumors into “lower-grade gliomas” (LGG), as opposed to the *IDH* wild type glioblastomas (GB), for the subsequent analyses [30–32]. As expected, LGG patients were younger than GB patients (mean age 46.4 vs. 62.7 years, *p* < 0.001).

**Table 1:** Characteristics of study patients

	Glioblastoma (*n* = 31)	LGG (*n* = 15)
Mean age (range)	62.7 years (19–81 years)	46.4 years (26–75 years)
*IDH* status	Wild type (*n* = 31)	Mutant (*n* = 15)
1p/19q status	Intact (*n* = 31)	Intact (*n* = 3)
		Codeleted (*n* = 12)
WHO grade	WHO IV (*n* = 31)	WHO II (*n* = 6)
		WHO III (*n* = 9)
PET	CT (*n* = 17)	CT (*n* = 6)
	MRI (*n* = 14)	MRI (*n* = 9)

### Hotspot volumes

**Fig. 1:**
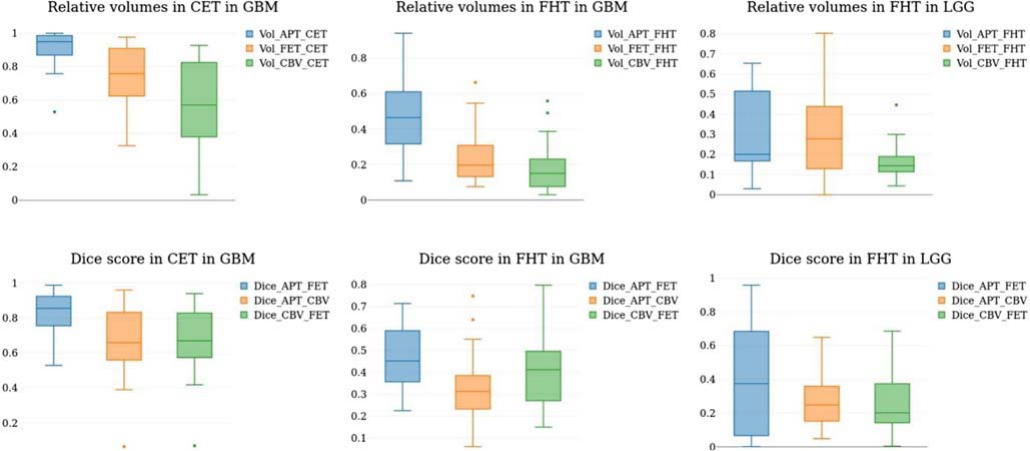
Volume and spatial measures. Boxplots of relative hot-spot volume (upper row, normalized by total volume) and Dice scores (lower row), separated for areas of contrast-enhancement in glioblastomas (left column) as well as FLAIR-hyperintense tumor in glioblastomas (middle column) and LGG (right column). Even in LGG, APTw hot-spots are observed. Note that across areas and entities, Dice overlap of APTw and FETw is higher than APTw/CBV and FET/CBV (lower row)

In LGG, only 5 out of 15 patients showed contrast-enhancement. Therefore, we only analyzed FHT in LGG. Median volume of FHT was similar in both entities (75,016 mm^3^ in GB vs. 55,496 mm^3^ in LGG, *p* = 0.21). Relative volumes of APTw, FET and CBV hotspots (normalized by the respective total volume) are shown in Fig. 1, upper row. Virtually the entire contrast-enhancing area of GB was APT-positive (median relative volume = 0.9495). This was significantly higher than both FET (median relative volume = 0.7577, *p* < 0.001) and CBV (median relative volume = 0.57079, *p* < 0.001). In the FHT in GB, the APTw-positive relative volume was also higher than both FET and CBV, albeit to a lower extent. Interestingly, we found only small APT-positive volumes in the FHT in LGG (median relative volume = 0.20112), significantly lower than in the FHT of GB (median relative volume 0.4656, *p* = 0.009515).

### Spatial overlap of imaging hotspots

**Fig. 2:**
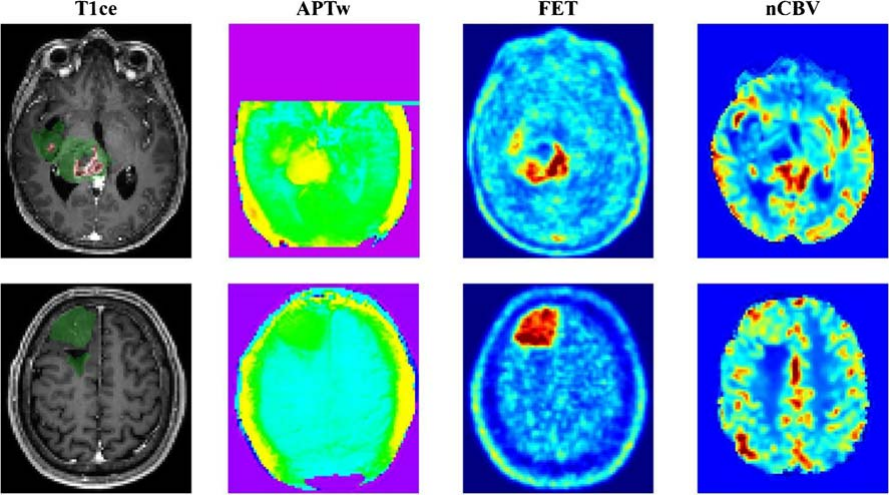
Example images of a GB (upper row) and LGG (lower row). Segmentation (CET in red, FHT in green) is overlaid on the contrast-enhanced T1 image (left-most column)

To further evaluate how well tumor areas defined by APTw, FET and CBV spatially overlap, we compared Dice scores (Fig. 1, lower row). We found that across entities and regions, the Dice overlap between APTw and FET was higher than both APTw/CBV and CBV/FET, especially in CET in GB. Here, the median Dice score between APTw and FET reached 0.8555. Interestingly, although APT-positive tumor volume was generally low in FHT in LGG, there still was an overlap between FET-positive and APT-positive areas (median Dice score = 0.37499). While the overlap between both APTw/CBV and FET/CBV was lower, we observed no clear trend for one pair overlapping more closely than the other. Figure 2 shows representative examples of a GB (upper row) and an LGG (lower row).

To assess if APTw and CBV are increased in FET-positive areas, we calculated median APTw and CBV values both in the entire CET and FHT as well as in FET hotspots in these areas (Table 2). While we did not observe a significant increase of APTw or CBV in the CET of GB given the already high Dice overlap in this area, we found that in the FHT in GB, both APTw and CBV were significantly (*p* < 0.001, respectively) higher in PET-positive areas. Even in LGG, we observed a similar trend for the two imaging modalities (*p* = 0.08476 for APTW and *p* = 0.0452 for CBV).

**Table 2:** Median and inter-quartile range values of APTw (a) and CBV (b) in CET and FHT, both in the entire area and only in those regions where FET-TBR is greater than 1.6

	Entire area	FET-positive (> 1.6)	*p* (Wilcoxon)
(a) APTw			
CET, GB	3.08 (2.79–3.72)	3.03 (2.74–3.69)	0.98
FHT, GB	1.79 (1.48–2.05)	2.42 (2.06–2.74)	< 0.001
FHT, LGG	1.35 (1.15–1.8)	1.67 (1.44–1.99)	0.08476
(b) CBV			
CET, GB	6.08 (4.6–9.46)	6.89 (5.24–9.82)	0.3
FHT, GB	2.24 (1.76–3.03)	4.59 (3.66–5.28)	< 0.001
FHT, LGG	2.95 (2.65–3.61)	3.86 (3.02–4.51)	0.04452

### Correlation of cellularity and vascularity with imaging

**Fig. 3:**
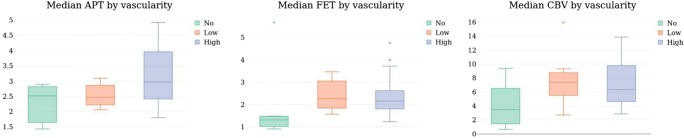
Imaging data and vascularity. Boxplots depicting the median APTw (left), FET (middle) and CBV (right plot) signal in biopsies without (green), low (orange), and high (blue) neovascularization

To better understand to what extent APTw, FET and CBV reflect key biological properties of gliomas, we compared cellularity and vascularity in 34 stereotactic biopsies of 10 glioblastoma patients (median 3 biopsies per patient, range 1–6). For all imaging modalities, we observed increasing values with higher vascularity of biopsies; however, this seemed less pronounced in APTw compared with CBV and FET (Fig. 3). In fact, the median values of both CBV (3.489) and FET (1.31) were below the cutoff for hotspots in areas without neovascularization, while also in these areas, median APTw was 2.521, i.e., above the cutoff. Conversely, we saw a significant correlation between APTw and cellularity (Spearman’s rho = 0.37, *p* = 0.02886) and a trend towards correlation between FET and cellularity (Spearman’s rho = 0.28, *p* = 0.11). For CBV, we observed no correlation with cellularity (Spearman’s rho = 0.11, *p* = 0.52).

## Discussion

In newly diagnosed gliomas, we found evidence for a relevant overlap of tumor areas defined by established cutoffs for APTw and FET, both in CET and FHT. This overlap was strongest in the contrast-enhancing areas of GB, but remained observable even in LGG, where APTw was generally low. Further speaking to the similar biological processes captured by APTw and FET, we found a correlation between cellularity for both imaging modalities.

Our findings contradict a recent report by da Silva et al. [18], who reported no overlap between APTw MRI and FET-PET in simultaneous PET-MRI of eight patients. APTw values cannot be directly compared, because different RF saturation (B_1,rms_ = 1.0μT, T_sat_ = 0.1 s) and image acquisition techniques (steady-state saturation transfer with 3D gradient echo EPI readout) were used in that study. The steady-state technique operates at a lower RF saturation duty cycle (74%) and part of the data (about 10%) is acquired with short saturation, before a steady-state is reached. Thus a lower APTw tumor to normal tissue contrast is expected. A low contrast-to-noise ratio (CNR) is further implied by using gradient echo readout with a low flip angle (11°) and spending only less than 10% of the acquisition time (70 offsets ± 14 ppm) near the nominal RF offset for APTw (+ 3.5 ppm). Higher CNR in shorter scan time is achieved with the fast spin echo sequence used in our study, spending 56% (5 out of 9 volumes) of the acquisition at + 3.5 ppm ± 0.8 ppm.

In contrast to that, our results agree with and expand several prior studies on APTw in LGG and GB [11, 12, 16]. In our study, the relative volumes of APT-hyperintense areas were significantly lower in the FHT in LGG compared with GB. This is in good agreement with the literature, where higher mean APTw values were found to be characteristic of more malignant gliomas. Nonetheless, by analyzing not just averaged voxel values, but integrating spatial information, we find that even in LGG, there are areas of APTw hyperintensity above the threshold defined by Jiang et al. [14]. More importantly though, even in LGG, we observe some overlap between APTw and FET. We speculate that areas defined as hotspots both by APTw and FET may represent the most malignant parts of tumors. This needs to be clarified in future prospective studies collecting targeted biopsies, guided both by FET and APTw.

A few studies have investigated how APTw and CBV can be used synergistically for tumor grading [12, 16] or differentiating tumor progression from pseudoprogression [33]. Across both entities and regions studied, we find that the overlap between APTw/CBV is relevantly lower than between APTw/FET, indicating a potential synergistic value of combining APTw and CBV information. This is further corroborated by investigating the stereotactic biopsies, where we find a more pronounced association of CBV and vascularity (compared with APTw) on the one side, and a stronger correlation of APTw with cellularity on the other side. Given the biological processes captured by both modalities, this disparity makes sense. As previously noted, we observe an association for FET with both cellularity and vascularity [34]. Our findings also underline the potential of multi-modal imaging to make oncogenic processes visible and thus support decision-making in clinically challenging situations such as glioma grading or differentiating tumor progression from radiation necrosis. Along this line, such information-rich datasets serve as an ideal basis for training machine learning classifiers which are able to (non-linearly) integrate the multimodal input data, as we and others have previously demonstrated [15].

While the cutoffs for FET [28] and CBV [29] are rather well-established, three studies have reported different thresholds for APT: for differentiating grade II from grade III/IV gliomas, Togao et al. [16] reported an APTw cutoff of 2.56 in 34 gliomas without intense contrast-enhancement. In contrast, Choi et al. [12] found a cutoff of 1.53 to optimize sensitivity and specificity in their setting. More recently, Jiang et al. reported their results on using APTw for distinguishing between vital tumor and radiation necrosis [14]: In a cohort of 21 patients with a suspected early tumor progression vs. pseudoprogression, several stereotactic biopsies were taken to account for intratumoral heterogeneity and a region-wise analysis of APTw intensities as performed. To maximize the discrimination between vital tumor and treatment effects, these authors report a cutoff of 1.79. For our analysis, we decided to apply this cutoff, as it best reflected active, vital tumor and was similar to the cutoff by Choi et al. However, we also investigated the increase of APTw values in PET-positive subregions of CET and FHT, eliminating the need for selecting an APTw cutoff. Given the almost perfect overlap of APTw and FET hot-spots in CET, we observed no significant differences in CET. Importantly though, we found significantly higher APTw values in PET-positive areas in FHT in GB and a strong trend towards higher APTw values in FET hotspots even in the FHT in LGG, where APTw was generally low.

In this study, we included PET acquisitions from PET-CT and PET-MRI. To minimize the potential bias when comparing PET data from both sources, we followed the approach from Koesters et al. [21], which demonstrated a good agreement between PET data from MRI and CT. We specifically investigated whether there were differences between volume and overlap data between patients scanned at different machines, but found no evidence for this (all comparisons *p* > 0.2). While scanning all patients in the same machine makes for a more homogeneous cohort, results obtained from images acquired on multiple scanners tend to be more robust and generalizable.

The main limitation of our study is the non-simultaneous acquisition of PET and APTw/CBV. While we aimed to minimize the delay, some cases had 4–5 days between both scans. Given the rapid growth and genomic instability of tumors, this may have an impact on the spatial overlap of imaging sequences. On the other hand, for biological imaging to be clinically useful (e.g., for biopsy or radiotherapy planning), the information contained in the images must be considered stable over a few days. In addition, the sample size investigated here is rather small. However, we took care to only include genomically well-characterized, accordingly grouped samples [19] to avoid leveling differences between biologically distinct groups of tumors. Nonetheless, validation of our findings in independent cohorts is necessary. Lastly, the scoring system for neovascularization used in our study is semiquantitative, and all biopsy samples were analyzed by a single neuropathologist to avoid interobserver variance, as described previously [34].

## Conclusions

Both in *IDH* wild-type glioblastomas and *IDH* mutant lower-grade gliomas, we observed relevant overlap between tumor areas defined by different imaging modalities, strongest for APTw and FET in contrast-enhancing parts of glioblastomas, but also in the FLAIR-hyperintense region of lower-grade gliomas. Further, we find that both APTw and FET are correlated with cellularity, as opposed to CBV. Given that APTw and FET reflect partially overlapping biological information like cellular density [11, 28], this is biologically plausible. Future work to better understand the complementary and synergistic value of biological imaging modalities is warranted.
